# Mucinous Carcinoma of the Skin: A Case Report

**DOI:** 10.31729/jnma.7415

**Published:** 2022-04-30

**Authors:** Adarsh Kumar Jhunjhunwala, Dilasma Gharti Magar, Dipesh Upreti, Niku Thapa, Arnab Ghosh, Sushma Thapa, Sudeep Regmi, Bishowdeep Timilsina

**Affiliations:** 1 Department of Pathology, Manipal College of Medical Sciences, Pokhara, Kaski, Nepal; 2 Manipal College of Medical Sciences, Poichara, Kaski, Nepal; 3 Department of Surgery, Manipal College of Medical Sciences, Pokhara, Kaski, Nepal

**Keywords:** *case reports*, *mucinous carcinoma*, *Nepal*, *sialomucins*

## Abstract

Primary mucinous carcinoma of the skin is a rare malignant neoplasm showing predilection to the periorbital region. These tumours are indolent and low-grade, with a tendency for local, sometimes multiple, recurrences. Distinguishing between these primary neoplasms and the more frequent metastatic mucinous deposits on the skin from primaries in the breast and gastrointestinal tract constitutes a diagnostic dilemma. In this case report, we have put forth the findings of a 70-year-old male who presented with a slow-growing periorbital swelling and was subsequently diagnosed with mucinous adenocarcinoma. An extensive workup in search of another primary tumour failed to show a primary malignancy elsewhere and the diagnosis of primary mucinous adenocarcinoma of the skin was rendered.

## INTRODUCTION

Mucinous Carcinoma of the Skin (MCS) was first described in 1952^1^ and later designated in 1971.^2^ It is an extremely rare entity with only about 380 cases reported in the literature.^3^ In a study that reviewed 73 such cases found a median age at onset of 63 years and male predominance of 2:1.^4^ Approximately 40% of primary MCS occurred on the eye of which 50% occurred on the lower eyelid. Though it is a carcinoma, the prognosis is relatively good, and metastasis to regional lymph nodes is quite rare. However, it is often destructive locally, and it sometimes recurs.^5,6^

## CASE REPORT

A 70-year-old male patient presented at the surgical out-patient department in March, 2021 with a complaint of periorbital swelling for 8 years. The patient complained of an increase in size and pain due to periorbital swelling for the past week. There was no history of associated fever, eye discharge, or trauma.

On clinical examination, the swelling was seen on the right periorbital region and was 1 cm below the lower eyelid, 2 mm away from the medial epicanthal fold. The swelling measured 3.5x2 cm on the lower eyelid and 6x8 cm over the lateral part of the right eye.

**Figure 1:**
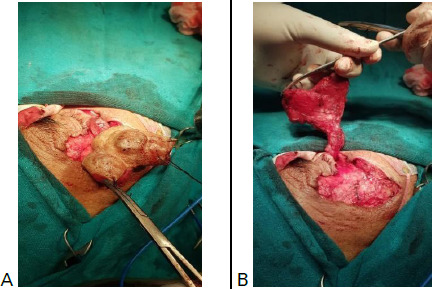
A) An intraoperative image of the mass shows its periorbital location and nodular appearance, B) Shows the extent of the lesion.

On ultrasonography, a lobulated outlined soft tissue density lesion in the subcutaneous plane of the right face region in the right periorbital region was seen. On colour and power doppler the lesion showed areas of vascularity with low flow. The underlying bones showed minimal scalloping. A radiological impression of lymphangioma was made after which complete excision of the tumour was planned (Figure 1).

**Figure 2:**
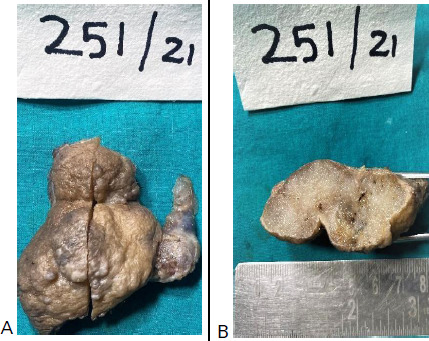
A) Gross images of the excised mass show a skin-covered nodular lesion, B) A cut section through the mass shows honeycombed spaces filled with gelatinous material.

After excision, the tumour was sent for histopathological evaluation. On gross examination, the specimen consisted of a skin-covered nodular tissue measuring 8x5.5x1.5 cm. The largest nodule measured 2.5x1.5 cm and the smallest nodule measured 2x1.5 cm. The cut section showed small honeycombed spaces filled with gelatinous material (Figure 2).

**Figure 3:**
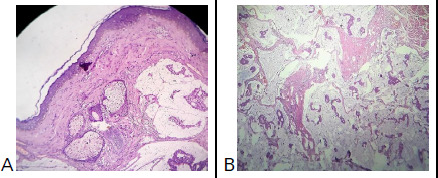
A) Microscopic image shows a tissue lined by keratinized stratified squamous epithelium with subepithelium showing lakes of mucin with floating tumour cells, B) A higher magnification shows floating tumour cells in the mucinous lakes.

Margins and base were identified by the operating surgeon. On microscopic examination, the tissue was seen lined by keratinized stratified squamous epithelium. Sub-epithelium showed lakes of mucin separated by delicate fibrous septae. Floating in between the mucinous lakes were nests, clusters, and cords of predominantly uniform round to cuboidal epithelial cells that showed a moderate amount of eosinophilic cytoplasm and mild to moderate nuclear variability. Focal areas with duct formation were noted. Occasional tumour nests showed prominent proliferation of cells in the cribriform pattern. The tumour showed no connection to the overlying skin or adnexal structures. The tumour was seen infiltrating into the underlying muscles. No areas of necrosis, mitosis or lymphovascular emboli were seen. All the margins and base showed infiltration by tumour cells except for lateral and inferior margins which were uninvolved. On staining with Periodic Acid-Schiff (PAS) and PAS with diastase, the mucinous pools retained the staining (Figure 3).

A diagnosis of mucinous carcinoma was made and the patient underwent exhaustive magnetic resonance imaging and computed tomography scans in order to look for secondaries. No secondaries were found on imaging and a diagnosis of primary mucinous carcinoma was made.

## DISCUSSION

Primary eccrine mucinous adenocarcinoma of the skin is a rarely described pathological entity. Mucinous carcinoma rarely originates in the skin; the majority of examples in the skin are actually metastatic to it.^5^ Common sites of origin of mucinous carcinomas are the breast, gastrointestinal tract, salivary glands, lacrimal glands, nose, paranasal sinuses, bronchi, renal pelvis, and ovary.^5^ Although no microscopic finding or immunohistochemical tool can distinguish MCS from mucinous carcinoma metastatic to the skin, this distinction is important because the prognosis of mucinous carcinoma metastatic to the skin is poorer than that of MCS. In our case, no primary tumour was detected in the breast, digestive tract, salivary glands, lacrimal glands, paranasal sinuses, lung, or kidney. Additionally, the absence of gastrointestinaltype epithelial cells, as well as the presence of diastases resistant mucin in the patient's tumour makes metastasis from a primary lesion in the colon or rectum, unlikely.^7,8^

The histogenesis of this tumour is still controversial. Some studies employing electron microscopy showed that the neoplastic cells of MCS were shown to be similar to the dark cells of normal eccrine glands.^9-11^ On the other hand, there have been reports suggesting that MCS have apocrine-type differentiation. A case report of MCS adjacent to a trichofolliculoma suggested that some cases of MCS originated from the follicle-sebaceous-apocrine unit.^12^ Another study suggested that MCS undergo apocrine differentiation as 1) Decapitation secretion is often observed in the neoplastic cells along the luminal border of tubules, 2) Identical features in MCS and colloidal carcinoma of the mammary gland, which is apocrine in origin, 3) Presence of plasmacytoid cells, which are found only in neoplasms with apocrine-type differentiation.^5^

The presentation of these neoplasms in the form of ulcers or cysts have also been described.^13^ The tumours usually range in size between 1 and 8 cm,^14^ the mean diameter prior to excision is reported to be around 1.8 cm.^15^ However, larger variants have been described in the literature.^14^ The nodules are well-circumscribed, unencapsulated, and often fixed to the dermis making them unable to be "shelled out". The cut surface is mostly gelatinous. Grossly, in the present case, the tumour was nodular and measured 8x5.5x1.5 cm. The cut section showed small honeycombed spaces filled with gelatinous material.

On histopathological evaluation, most tumours show no connection to the overlying epidermis or skin adnexa. Typically, there are pools of basophilic sialomucin with floating tumour cells compartmentalised by delicate thin fibrous septae creating a honeycomb pattern. The neoplastic cells are round to cuboidal with abundant eosinophilic to clear cytoplasm, small central nuclei, minimal nuclear pleomorphism, and only rare mitotic figures. These tumour cells are often grouped in nests and duct-like structures reminiscent of eccrine and apocrine sweat glands. Occasional glandular structures may show prominent proliferation and form a cribriform pattern.^8,13^ The presence of copious amounts of sialomucin has been hypothesised to serve as a physical barrier to spread by compressing the tumour stroma and limiting growth, as well as impeding DNA synthesis, declining the rate of angiogenesis, and subsequently metastatic disease.^13,16^

The rate of local recurrence after excision with narrow margins is as high as 30%. Lesions on the eyelid, especially the inner canthus, have the highest recurrence rate. The rate of local metastasis to regional lymph nodes has been reported as 10%. Because of this recurring nature of the tumour, adequate excision with wide margins (at least 1 cm) is advocated. This is especially hindered by the periorbital location of the tumour. Moh's micrographic surgery can be principally a valuable treatment modality. These tumours are generally resistant to radiotherapy and chemotherapy and these tools are not usually employed.^8,13^ In the current case, the tumour was only 2 mm away from the medial epicanthal fold, due to which wide excision with a 1 cm margin was not possible.

Primary mucinous carcinoma is a rare entity with an indolent course. Due to limited information and the applicability of immunohistochemistry, a thorough oncological evaluation must be carried out to rule out metastasis from other sites. Traditional histochemical stains for mucin-like Alcian blue and PAS combined with diastase can help rule out primarily the salivary gland and gastrointestinal tumours. Further, patients must be educated about the diagnosis, and indolent nature of the tumour and counselled about the importance of regular follow-up to rule out local tumour recurrence or the development of regional lymphadenopathy.
